# Innocent Amusements

**Published:** 1859-10

**Authors:** 


### INNOCENT AMUSEMENTS.

Looking at a little news-boy trying to cry the papers when his face is half stiffened with cold.

Contemplating the movements of a street sweeper when working by the day.

Witnessing the solemn phiz and happy faith of a homœopathic rinsing and wiping a bright silver teaspoon to take its fill of cold water from a glass, alternating with another spoon and glass.

Noticing an effort at laughter after having tasted a green persimmon.

Contemplating the predicament of a doctor when, on expressing his delight at the speedy recovery of his patient, he is informed that his medicine was not taken.

Beholding the fall from gay to grave, in the person of a highly imaginative lady on being informed that the remedy which she had just praised so highly for its wonderful efficacy in curing her last ailment was a——bread pill.

